# How large is the burden of depression in a medical school? A cross-sectional study among medical students in Nigeria

**DOI:** 10.11604/pamj.2021.40.71.29079

**Published:** 2021-10-01

**Authors:** Edmund Ndudi Ossai, Irene Ifeyinwa Eze, Rejoice Chinecherem Onyenakazi, Elias Ugebe, Basil Eze, Obinna Obasi

**Affiliations:** 1 Department of Community Medicine, College of Health Sciences, Ebonyi State University, Abakaliki, Nigeria,; 2 Department of Community Medicine, Alex Ekwueme Federal University Teaching Hospital, Abakaliki, Nigeria

**Keywords:** Depression, medical school, medical students, Beck’s depression inventory, Ebonyi State, Nigeria

## Abstract

**Introduction:**

mental health of students deteriorate when they begin studies in a medical school. The study aimed to determine the prevalence of depression and associated factors among medical students in a university in Nigeria.

**Methods:**

a cross-sectional study design was used. All matriculated medical students of Ebonyi State University Abakaliki, Nigeria were included in the study. Information was obtained using a self-administered structured questionnaire. Beck´s depression inventory was used to determine the prevalence of depression. Chi square test was used to ascertain association between variables. Level of statistical significance was determined by p value of <0.05.

**Results:**

the mean age of the students was 23.2 ± 3.3 years and majority, 60.2% were males. Prevalence of depression was 17.4%. Third-year class (major examination class) had the highest proportion of students who were depressed, 24.2% while final year class had the least, 8.8%. Coping mechanisms for low moods included sleeping, 50.4% and having interactions with colleagues, 46.9%. Factors associated with depression included being <25 years, (p=0.008), being in the pre-clinical school, (p=0.023) and being afraid some students may not graduate from medical school (p=0.030).

**Conclusion:**

burden of depression was high among the students and most pronounced among third year students. There is need for proper orientation of newly admitted medical students on the medical curriculum by authorities of the university. Efforts should be made to decrease undue anxiety among students especially during examination. Adequate measures should be put in place for early detection and prompt management of cases of depression among the students.

## Introduction

According to the World Health Organization (WHO), mental diseases will constitute the highest disease burden globally by the year 2030 [1]. There is evidence that mental health of students deteriorates when they begin studies in a medical school and remains poor throughout the period of training [2]. Depression has been acclaimed to be the second most prevalent condition worldwide in the year 2020 hence it is regarded as one of the commonest mental health illness [3]. Studies have revealed that the prevalence of depression is higher among medical students than their peers in other faculties [4,5]. The findings of systematic reviews revealed that the prevalence of depression among medical students were 27.2% [6] and 27% [7]. This high prevalence notwithstanding, psychological problems like academic stress and mental health states like depression among medical students have been found to be under diagnosed and under-treated [8].

It has been found that perceived stress is related to increased levels of depression [9]. A study in a medical school in Nigeria revealed that the prevalence of stress among medical students is very high [10]. This supports the need for studies on prevalence of depression among medical students. Also, medical students are known to be less likely than the general population to identify symptoms of depression or access treatment for the disease when the need arises [11] in spite of the assumption that they have good access to health care. Thus, it has been postulated that mechanisms to facilitate early detection including prevalence studies, counselling services and treatment of depression among medical students should be provided in medical schools [12].

Equally important is the need to quantify the burden of depression and factors associated with it among medical students so as to enhance their counselling and efforts toward rehabilitation [13,14]. Suffice it to say that medical students of today are the physicians of tomorrow hence the need to pay attention to issues related to their mental health. This is of relevance as depression has adverse consequences including suicide, school dropout and drug abuse [15]. It also leads to burnout [16] which is capable of affecting the relationship between the doctor and patient. Furthermore, there is evidence that depression negatively affects quality of life [17,18]. This study was designed to determine the prevalence of depression and associated factors among medical students of Ebonyi State University Abakaliki, Nigeria.

## Methods

**Study setting:**the medical school of Ebonyi State University Abakaliki, Nigeria was established in 1999 and was one of the pioneer faculties of the university. The university admits an average of one hundred students each year based on the recommendation of the Medical and Dental Council of Nigeria. In all medical schools in Nigeria, the study of medicine is for a period of six years with each of the years regarded as levels. Academic activities in the first year (100 level) also called the preliminary year take place at the faculty of science, the second and third years (200 and 300 level) are regarded as the pre-clinical years while the fourth to sixth years (400 to 600 level) constitute the clinical period of training. The students receive their clinical training at Alex Ekwueme Federal University Teaching Hospital Abakaliki, Nigeria.

**Study design and participants:** the study employed a whole population cross-sectional study design. The population eligible for study included all matriculated medical students at the College of Health Sciences of Ebonyi State University Abakaliki, Nigeria. Medical students who were not present during the period of study and who did not give consent to participate were excluded from the study. Five hundred and twenty-two students took part in the study representing a response rate of 87.0%.

**Study instrument:** the study instrument included a validated questionnaire, the Beck´s Depression Inventory (BDI) [19]. It consists of 21 questions with a four-point Likert scale ranging from 0-3 (Annex 1). A score of zero to each of the variables in the questionnaire meant not at all, a score of one represented sometimes while a score of two meant most of the time. A response of all the time was recorded as three. The scoring system used in this study was as recommended by the originators of the inventory. The maximum possible score for any respondent is 63 while the lowest possible score is zero. The total score for each respondent was determined by adding the score obtained by each respondent for the 21 questions in the questionnaire. From the total scores of the respondents, the level of depression is classified as: 1-10: these ups and downs are considered normal; 11-16: mild mood disturbance; 17-20: borderline clinical depression; 21-30: moderate depression; 31-40: severe depression; over 40: extreme depression. The other components of the questionnaire included a section on the sociodemographic characteristics of the students and how the students coped with low moods. These aspects of the questionnaire were developed by the researchers and pretested before use. The questionnaire was self-administered.

**Ethical consideration:** ethical approval for the study was obtained from the Research and Ethics Committee of Ebonyi State University Abakaliki. (EBSU/DRIC/UREC/Vol. 04/064). The students were required to sign a written informed consent before participating in the study and the nature of the study, its relevance and the level of their participation were made known to them. Participation in the study was voluntary. Respondents were assured that all information provided through the questionnaire will be kept confidential. Also, the name of the respondents was not included in the questionnaire.

**Data management:** data entry and analysis were done using IBM Statistical Package for Social Sciences (SPSS) version 25. Frequency tables and cross tabulations were generated. Chi square test of statistical significance was used in the analysis and the level of statistical significance was determined by a p value of <0.05. Outcome measure of the study is the proportion of students who were depressed using the Beck´s depression inventory and the factors associated with depression among the students.

## Results

Table 1 shows the sociodemographic characteristics of the respondents. The mean age of the respondents was 23.2 ± 3.3 years and majority of the respondents, 53.6% were in the age range 20-24 years. Majority of the respondents, 60.2% were males. The highest proportion of the respondents, 19.7% were in 400 level class while the least proportion, 13.6% were in 300 level class. Table 2 shows the prevalence of depression among the respondents. Among the respondents, 17.4% were depressed. The highest proportion of the respondents who were depressed, 24.2% were in the 300-level class while the least proportion, 8.8% were in the final year class.

**Table 1:** socio-demographic characteristics of medical students in Nigeria

Variable	Frequency (n=522)	Percent
**Age of respondents**		
**Mean (±SD)**	**23.2 ± 3.3**	
**Age of respondents in groups**		
<20 years	76	14.6
20 - 24 years	281	53.8
25 - 29 years	144	27.6
≥30 years	21	4.0
**Gender**		
Male	314	60.2
Female	208	39.8
**Level of study**		
100 level	80	15.3
200 level	91	17.4
300 level	71	13.6
400 level	103	19.7
500 level	92	17.6
600 level	85	16.3
**Marital status**		
Single	499	95.6
Married	23	4.4
**Religion**		
Christianity	516	98.9
Islam	4	0.8
Traditional religion	2	0.4
**Ethnicity**		
Igbo	511	97.9
Yoruba	6	1.1
Hausa	4	0.8
Minority tribes	1	0.2

**Table 2:** prevalence of depression among medical students in Nigeria

Variable	Frequency (n=522)	Percent
Levels of depression		
Normal	383	73.4
Mild mood disturbance	48	9.2
Borderline clinical depression	60	11.5
Moderate depression	22	4.2
Severe depression	7	1.3
Extreme depression	2	0.4
**Prevalence of depression**		
Yes	91	17.4
No	431	82.6
**Prevalence of depression by academic levels**	**(n=91)**	
100 level	17	18.7
200 level	15	16.5
300 level	22	24.2
400 level	16	17.6
500 level	13	14.3
600 level	8	8.8

Table 3 shows the coping mechanisms for low moods among the respondents. The highest proportions of the respondents cope with low moods by sleeping, 50.4%, discuss with friends/classmates, 46.9% and by wishing it away, 32.2%. Table 4 shows the factors associated with depression among the respondents. A significantly higher proportion of respondents who were less than 25 years of age, 20.4% were depressed when compared with those who were 25 years and above, 10.9%, (χ^2^=7.134, p=0.008). The highest proportion of the respondents who were depressed were in the preclinical school, 22.8% while the least proportion, 13.2% were in the clinical school and the difference in proportion of respondents who were depressed was found to be statistically significant, (χ^2^=7.562, p=0.023). Also, a significantly higher proportion of the students who were afraid some students may not graduate from medical school, 22.0% were depressed when compared with those were not afraid, 14.6% (χ^2^=4.698, p=0.030).

**Table 3:** coping mechanisms for low moods among medical students in Nigeria

Variable	Frequency (n=522)	Percent
Coping strategies for low mood**		
Sleep	263	50.4
Discuss with colleagues	245	46.9
Wish it away	168	32.2
Inform parents/relatives	158	30.3
Eat repeatedly	105	20.1
Crying	84	16.1
Option for sex	77	14.8
Discuss with priests/pastors	76	14.6
Drink more alcohol	56	10.7
Leave school for a moment	34	6.5
Use of stimulants	18	3.4
Smoke cigarettes	14	2.7

** multiple responses

**Table 4:** factors associated with depression among medical students in Nigeria

Variable	Depression among medical students (n=522)		χ2	p value
	Yes n (%)	No n (%)		
**Age of respondents**				
<25 years	73 (20.4)	284 (78.6)	7.134	0.008*
≥25 years	18 (10.9)	147 (89.1)		
**Gender**				
Male	49 (15.6)	265 (84.4)	1.829	0.176
Female	42 (20.2)	166 (79.8)		
**Stage of training**				
Preliminary year	17 (21.3)	63 (78.8)	7.562	0.023*
Preclinical	37 (22.8)	125 (77.2)		
Clinical	37 (13.2)	243 (86.8)		
**Has an academic adviser**				
Yes	39 (15.7)	209 (84.3)	0.957	0.328
No	52 (19.0)	222 (81.0)		
**Marital status**				
Single	86 (17.2)	413 (82.8)	0.310	0.578
Married	5 (21.7)	18 (78.3)		
**Afraid some students may not graduate from medical school**				
Yes	44 (22.0)	156 (78.0)	4.698	0.030*
No	47 (14.6)	5.4)		

* Statistically significant; χ2: Chi square test

## Discussion

The prevalence of depression among the students was 17.4%. This proportion has huge implications on the life of medical students and thus the burden could be regarded as high. Similar proportions were reported in a study among two medical schools in southeast Nigeria [20] and in a study in Vietnam [21]. A higher proportion of students than was obtained in this study were found to be depressed in other studies involving medical students in Africa [22-24] as well as other studies outside Africa [25-30]. Also, higher proportion of medical students were reported depressed from systematic reviews [6,7]. Based on these observations, a study concluded on the importance of initiating stress management strategies among medical students as a way of preventing depression among the students [4]. Also, the need to support the students during the period of training has been suggested through the provision of counselling services so as to detect and manage cases of depression as early as possible [12].

The highest proportion of the respondents who were depressed were in the third year of their study, followed by first year students, while the least proportion were in the final year class. The third year remain the most critical in the life of the medical student in Nigeria as it is the year of the first professional examination for the students. Based on this, the result is to an extent expected. Perhaps, this explains why a similar result was obtained from another medical school in southeast Nigeria [22]. This observation is however different from what was observed in other countries where the second-year students had the highest proportion of those who were depressed [25,27]. In another study, the first-year students had the highest proportion of those who were depressed [31]. It is important to note that the numbering of the study years for medicine in Nigeria may be different from what is obtained in these countries.

From the results of this study, the proportion of respondents who were depressed in the first year of study when compared with the other years was high and rather surprising. This is because the course content in the first year regarded as the preliminary year in Nigerian medical schools is light when compared with the other years. Moreover, it could be expected that the students in the first year are in joyful mood bearing in mind the intense competition to gain admission to study medicine in Nigeria. However, this finding may be explained by the fact that first year is also still regarded as a screening year and may have caused anxiety and mood changes for the students, hence the high depression rate. This observation necessitates that newly admitted medical students are properly oriented on the medical curriculum by officers of the faculty and the university. This will give the students first-hand information of what is expected from them in terms of life as a medical student rather than obtaining such from other students or any other person in which case information may be distorted.

The results of this study reveal that majority of the students, 73.4% were normal. Only 4.2% had moderate depression while the least proportion, 0.4% had extreme depression. In a study in India, majority 69.9% were adjudged normal, 6.4% had moderate depression while the least proportion, 0.7% had very severe depression [28]. The result from a study in Malaysia is however different in which 45% were normal while 13.5% had moderate depression [30]. In another study in Egypt, 19.5% were normal while 24.4% had moderate depression [5]. This is an indication that different countries have varied burdens of depression among their medical students. Suffice it to say that depression among medical students remain a general issue. This necessitates that individual medical schools may have a good role to play in ensuring the well-being of the students including instituting counselling services for the students.

The coping strategies for low mood among the students included sleeping 50.4%, interacting with colleagues, 46.9% and wishing it away, 32.2%. In another study in a medical school in southeast Nigeria the strategies included coping passively, 25% and discussing with friends and classmates, 23.9% [22]. In another study in Kenya, the coping strategies among the students included seeking help from peers, 90% and alcohol use 80% [32]. These observations bring to the fore the importance of support of classmates to the well-being of medical students during the period of training.

A significantly higher proportion of respondents who were less than 25 years of age were depressed when compared with those who were 25 years and above. Hence the younger the age group the higher the proportion of those who were depressed. This is similar to what was obtained in a study in Pakistan [25]. This finding is however at variance with what was obtained in a medical school in Egypt where higher depression scores were associated with increasing age [33]. It could be assumed that age may have a moderating effect on the experiences of the students during the training period in medical school.

The proportion of the respondents who were depressed was significantly higher in the preclinical years compared to the clinical years. In Nigeria, uncertainties about student progression in a medical school is more at the preclinical period of training. Thus, this may be an explanation for this finding. Consistent with our findings, from the result of studies in India and Bangladesh, significantly higher proportions of students who were in the first and second year of study were depressed when compared with those in higher levels [27,29]. Similarly, a study in Turkey reported that depression among the students decreased with increasing year of study [34]. Our finding is however at variance to what was obtained in Cameroon where the odds of being depressed were four times more with the clinical students when compared with those in the preclinical [23]. Also, results of a study in India reported that the proportion of first and second year medical students who were depressed were significantly lower than those in higher levels of study [28]. The fact that different countries may have different medical curricula and these curricula perceived differently by the students may explain these variations.

A higher proportion of the students who were afraid that some students may not graduate from medical school were depressed when compared with those who were not afraid. A study in Malaysia, revealed that a higher proportion of medical students who were under undue pressure during examinations were depressed when compared with those who were not [24]. Also, dissatisfaction with examination criteria was found to be associated with depression among medical students [25]. Similarly medical students who have undue concerns about the future were found to have a higher prevalence of depression when compared with those who had no such concern [26]. It has been postulated that a supportive academic staff is of immense relevance to medical students during the training period [35], as this will enhance the satisfaction of the students with their medical training. Medical students, when satisfied with their training have decreased odds of being stressed during the period of training [35]. This is in tandem with the suggestion to make the academic curriculum in medical schools more student friendly [36]. This is expected to reduce the stress during the medical training period which is one of the factors responsible for depression among the students.

**Limitation of the study:** the main limitation is that the study was carried in one institution. This may limit its generalizability. However, the study provided insight on the study group which is typical of many medical students in Nigeria and Africa, and consequently, results are likely to be generalized to other similar settings.

## Conclusion

The burden of depression was high among the students and most pronounced among third year students. There is need for proper orientation of newly admitted medical students on the medical curriculum by authorities of the university. Efforts should be made to decrease undue anxiety among the students especially during examination. Medical students preparing for major examinations should be well supported and encouraged by their colleagues. Adequate measures should be put in place for early detection and prompt management of cases of depression among the students.

### What is known about this topic

- Prevalence of depression among medical students is high;
- Prevalence of depression is higher in some levels of class in medical school compared to others;
- Different countries have varied burden of depression among their medical students.

### What this study adds

- Different countries have different medical curricula which may be perceived differently by the students and how it influences their health;
- Support from classmates to the well-being of medical students during the period of training is crucial;
- Depression among medical student is not only about content of the curriculum as class levels with light content (preliminary class) could have high prevalence.

## Annex

**Annex 1**: questionnaire on prevalence of depression (PDF-119Kb)
